# Protein functional domain analysis enhances genotype–phenotype associations in comparative genomic studies of *Pseudomonas aeruginosa*

**DOI:** 10.3389/fmicb.2025.1569118

**Published:** 2025-08-06

**Authors:** Irene Bianconi, Alfonso Esposito, Silvano Piazza, Elena Piffer, Elisabetta Pagani, Olivier Jousson

**Affiliations:** ^1^Department of Cellular, Computational and Integrative Biology - CIBIO, University of Trento, Trento, Italy; ^2^Laboratory of Microbiology and Virology, Provincial Hospital of Bolzano (SABES-ASDAA), Lehrkrankenhaus der Paracelsus Medizinischen Privatuniversität, Bolzano, Italy; ^3^Computational Biology Unit, International Centre for Genetic Engineering and Biotechnology, ICGEB, Trieste, Italy

**Keywords:** *Pseudomonas aeruginosa*, genotype–phenotype relationship, antimicrobial resistance, comparative genomics, functional domains

## Abstract

**Introduction:**

*Pseudomonas aeruginosa (P. aeruginosa)* represents a paradigm for studies on antibiotic resistance. Nevertheless, despite the considerable number of genome sequences that have been released in recent years, there is still a paucity of knowledge regarding the genomic determinants of the typical phenotypic traits associated with pulmonary infection.

**Methods:**

The genomes of 40 strains of *P. aeruginosa* were sequenced over an 8-year period (2007–2014), isolated from the sputum of a single patient with cystic fibrosis in Trentino, Italy. The same isolates were characterised for a panel of 14 phenotypes, including biofilm formation, antibiotic resistance, secretion of siderophores and virulence factors. The phylogenetic coherence of the measured phenotypes was determined in relation to the tree based on single-nucleotide polymorphisms (SNPs). Subsequently, the semantic framework for comparative functional genomics (SAPP) was employed to investigate the depletion or enrichment of specific protein functional domains within the population in relation to the observed phenotypes.

**Results:**

The majority of our findings regarding phenotypic adaptation over time were consistent with the population structure and followed the evolutionary pathways described in the literature. However, an exact relationship between the presence of genes and specific phenotypes could not be established. The SAPP approach enabled the identification of 189 protein domains that were significantly enriched in antibiotic-resistant strains, as well as 87 domains associated with other phenotypic adaptations. In some cases, the domains were commonly associated with antibiotic resistances, for example, outer membrane efflux pumps and porins. However, we also detected a number of domains with unknown function.

**Discussion:**

Our findings provide a foundation for a more comprehensive understanding of the phenotypic adaptations occurring during microevolution in lung environments and facilitate the identification of new targets for the design of novel antimicrobial agents.

## 1 Introduction

Antimicrobial resistance (AMR) constitutes a critical and escalating global health concern, fuelled by complex epidemiological dynamics. The spread of antibiotic-resistant microorganisms increasingly undermines the efficacy of current therapeutic regimens, posing a substantial threat to human health (O’Neill, 2016; Murray et al., 2022).

*Pseudomonas aeruginosa (P. aeruginosa)* has emerged as a model organism for the study of antimicrobial resistance (AMR; Oliver et al., 2024), including low outer membrane permeability, constitutive expression of multidrug efflux systems, and the production of antibiotic-inactivating enzymes (Breidenstein et al., 2011; Elfadadny et al., 2024). In particular, Cystic Fibrosis (CF) patients, with chronic colonisation with *P. aeruginosa*, are exposed to increased morbidity and mortality (Bhagirath et al., 2016; Rossi et al., 2021; Cramer et al., 2023); estimates suggest that 25–45% of adult CF patients are chronically infected with multidrug-resistant (MDR) *P. aeruginosa* strains within their airways (Lechtzin et al., 2006), a rate, however, that has been observed to be on the rise, since MDR *P. aeruginosa* strains are developing with increasing frequency and rates have tripled in the last two decades (Kothari et al., 2023). It is a matter of concern that CF strains of *P. aeruginosa* have already been found to be resistant to nearly all or all antibiotics in clinical use today. The microbiology of pulmonary infections in CF patients is often different from similar infections in healthy individuals (Folkesson et al., 2012).

Antibiotic resistance is often associated with other phenotypic adaptation such as biofilm formation and hypermutability. Biofilm formation plays a central role in chronic infections, providing a protective matrix that limits antibiotic diffusion and impairs host defences. Biofilm formation is regulated by the *las* and *rhl* quorum sensing system (Davies et al., 1998; Vetrivel et al., 2021). Within biofilms, *P. aeruginosa* cells are embedded in a self-produced extracellular matrix composed of polysaccharides, proteins, and extracellular DNA. This lifestyle leads to reduced metabolic activity and altered gene expression, resulting in increased tolerance to antimicrobial agents (Ciofu and Tolker-Nielsen, 2019; Reichhardt, 2023). Furthermore, biofilm-growing bacteria quickly become MDR (Bjarnsholt et al., 2009; Metzger et al., 2022). Hypermutable strains, meanwhile, accelerate the accumulation of mutations that can enhance survival under selective pressure. The prevalence of hypermutable *P. aeruginosa* isolates has been documented approximately of 5–10% at onset/early colonisation in CF patients (Kenna et al., 2007; Mena et al., 2008).

Strategies to mitigate the emergence and dissemination of MDR pathogens include optimised antibiotic stewardship, improved diagnostic and surveillance frameworks, and the development of novel antimicrobial agents (Roca et al., 2015). Despite the urgent need for new antibiotics, the discovery pipeline remains constrained; since 2000, only a limited number of newly approved antibiotics have introduced novel classes or mechanisms of action (Renwick et al., 2016; Butler et al., 2024). Consequently, innovative approaches are required to identify new therapeutic targets.

MDR and other phenotypic adaptations can be associated to common genetic determinants, which can be tracked by whole genome sequencing (WGS). WGS has become an essential tool for understanding the microevolutionary trajectories and identifying new targets for the development of antimicrobial therapies (WHO, 2020; Bianconi et al., 2023). Bacterial WGS allows the query of complete genetic repertoire and the discovery of yet uncharacterised determinants (Köser et al., 2014; Chan, 2016; Waddington et al., 2022). Nevertheless, knowledge on genome microevolution during persistent CF infection and the genomic features associated with the phenotypic adaptation in CF patient lung environment is incomplete; therefore, it is very likely that many of the genes involved are still to be identified. Genotype–phenotype mapping remains one of the most challenging issues in understanding bacterial microevolution and adaptation (Manrubia et al., 2021). Comparative genomics enables the identification of strain-specific adaptations, genomic islands, and horizontally acquired elements that contribute to virulence, persistence, and antimicrobial resistance. Functional domain analysis, on the other hand, focuses on conserved protein motifs (e.g., PFAM domains) that are often preserved across diverse genetic backgrounds. This approach analysis enables detection of conserved protein motifs across diverse genetic contexts, even in hypothetical or poorly annotated genes. This is crucial in *P. aeruginosa*, where phenotypic traits such as antibiotic resistance or biofilm formation often arise from complex, polygenic networks. By associating specific domains with phenotypic traits, we are able to gain insight into the functional architecture of adaptation, highlighting novel targets for therapeutic intervention.

Traditional target identification strategies, which rely on mutational analysis of individual genes, are often limited in scope and efficacy. On the other hand, advances in WGS approaches and computational biology offer the potential to revolutionise drug discovery strategies and approaches. Nevertheless, despite the increasing availability of genome sequences, the functional interpretation of genotypic variation in relation to phenotype remains limited.

In this study, we applied a semantic framework for comparative functional genomics to associate conserved protein domains with phenotypic traits in a longitudinal collection of clonal *P. aeruginosa* isolates from a CF patient, aiming to identify potential drivers of adaptation and resistance. Our innovative approach entails a shift in focus from gene counting to the quantification of protein domains within the genome, given that these domains are proportional to the number of protein-coding genes. This method addresses the challenges posed by uncharacterised or unannotated genes, as protein domains can be reliably identified through PFAM databases (Mistry et al., 2021). The identification of multiple functional domains associated with each gene results in the generation of a greater number of features, thereby facilitating the inference of correlations with specific phenotypes. In contrast to traditional methods, which may fail to identify key targets, this comprehensive approach allows for the precise localisation of all potential targets within the bacterial genome, including those involved in the persistence and antibiotic resistance of *P. aeruginosa*, the roles of which remain unknown.

## 2 Methods

### 2.1 Bacterial strains

Over an eight-year period (2007–2014), 40 strains of *P. aeruginosa* were isolated from the sputum of a single male CF patient. The patient was treated at the Trentino Regional Support CF Centre and the Operative Unit of Clinical Pathology (Rovereto Hospital, Trentino, Italy). The patient was 24 years of age at the commencement of sampling. The patient is heterozygous for the CFTR mutations ∆F508 and G542X (Bianconi et al., 2016, 2019; Table 1).

**Table 1:** Clonal strain collection with isolation date and sequence type.

Isolate name	Isolation date	Sequence type
TNCF_3	13/04/2007	390
TNCF_4M		390
TNCF_6	29/05/2007	390
TNCF_7M		390
TNCF_10	26/07/2007	390
TNCF_10M		390
TNCF_12	4/9/2007	390
TNCF_13	5/9/2007	390
TNCF_14		390
TNCF_16	24/09/2007	1864
TNCF_23	17/10/2007	390
TNCF_23M		390
TNCF_32	23/11/2007	390
TNCF_32M		390
TNCF_42	31/01/2008	390
TNCF_42M		390
TNCF_49M	5/9/2008	390
TNCF_68	2/8/2010	390
TNCF_69	22/03/2010	1863
TNCF_76	9/6/2010	390
TNCF_85	8/11/2010	1864
TNCF_88M	14/12/2010	1864
TNCF_101	30/06/2011	1864
TNCF_105	25/08/2011	390
TNCF_106	16/09/2011	390
TNCF_109	30/09/2011	390
TNCF_130	19/07/2012	390
TNCF_133	14/09/2012	390
TNCF_133_1		1864
TNCF_151	05/04/2013	390
TNCF_151M		1864
TNCF_154	29/04/2013	390
TNCF_155	06/05/2013	390
TNCF_155_1		1923
TNCF_165	23/09/2013	1923
TNCF_167	3/10/2013	390
TNCF_167_1		390
TNCF_174	24/04/2014	390
TNCF_175	23/06/2014	390
TNCF_176	11/8/2014	1923

The strains are available at the Microbial genomics laboratory of the University of Trento.

### 2.2 Phenotypic characterisation

The siderophores secretion assay was performed using the protocol of Alexander and Zuberer (1991), in brief, we prepared M9 media complemented with casamino acid solution, piperazine diethansulfonic acid (PIPES) and 1 mM FeCl_3_. The strains were inoculated in this medium and incubated overnight at 37°, siderophore secretion was detected as an orange halo around the colonies. Pyocyanin production levels were determined by measuring the ratio of the OD_695_ of the supernatant divided by the respective OD_600_ in king A medium (Bianconi et al., 2011). The pyoverdine secretion was assessed by visual assay after a single colony was inoculated in 10 mL of LB broth (LB, Oxoid), and incubated for at least 24 h at 37°C (Marvig et al., 2013). To detect LasR mutants, the isolates were inoculated on LBA for 24 h at 37°C. Agar plates were left on the bench at RT for 1 to 3 weeks; then the plates were stained with 0.1% crystal violet. A metallic sheen indicated LasR mutants. The ability to lyse erythrocytes was measured plating each strain onto a blood agar plates (Kidma) and incubating for 48 h at 37°C. Haemolysis was indicated by an halo around the colony (Hoboth et al., 2009). To measure the resistance to Amikacin, Cefepime, Ceftazidime, Ciprofloxacin, Colistin, Doripenem, Fosfomycin, Gentamycin, Imipenem, Levofloxacin, Meropenem, Piperacillin/Tazobactam, we used the Sensititre GRAM Negative Plate Format (Thermo Scientific, Waltham, MA, United States); the complete methos was previously published by the authors (Bianconi et al., 2019). The mutation rate was measured using the rifampicin assay (Oliver et al., 2000), the biofilm production was assessed by the crystal violet staining assay with minor modifications (O’Toole, 2011). To measure swarming motility, single colonies were picked and inoculated on agar plates and incubated for 15 h at 37°C, after incubation the halo around the colony was measured (Bianconi et al., 2011). Protease production was evaluated using the skim milk assay, measuring the diameter of milk turbidity clearing surrounding each colony (Brown and Foster, 1970). For the phenotypes of pigmented colonies or mucoid phenotype we visually inspected on PIA (*Pseudomonas* Isolation Agar) plates. All assays were performed in triplicates.

### 2.3 Genome sequencing, assembly and annotation

The complete methodology for genome sequencing, assembly, and annotation of the 40 *P. aeruginosa* isolates was previously described in Bianconi et al. (2019). Briefly, genomic DNA was extracted using the DNeasy Blood and Tissue Kit (Qiagen), libraries were prepared with the Nextera XT DNA Library Preparation Kit (Illumina), and sequencing was performed on the Illumina MiSeq platform. *De novo* assembly was carried out using SPAdes v3.1.0, refined with Bowtie2, and reordered with Mauve. Genome annotation was performed using Prokka v1.11. SNPs were identified using Snippy, and phylogenetic analysis was conducted with BEAST.

### 2.4 Sequence data can be retrieved from Genbank (BioProject PRJNA326244)

**Figure 1:**
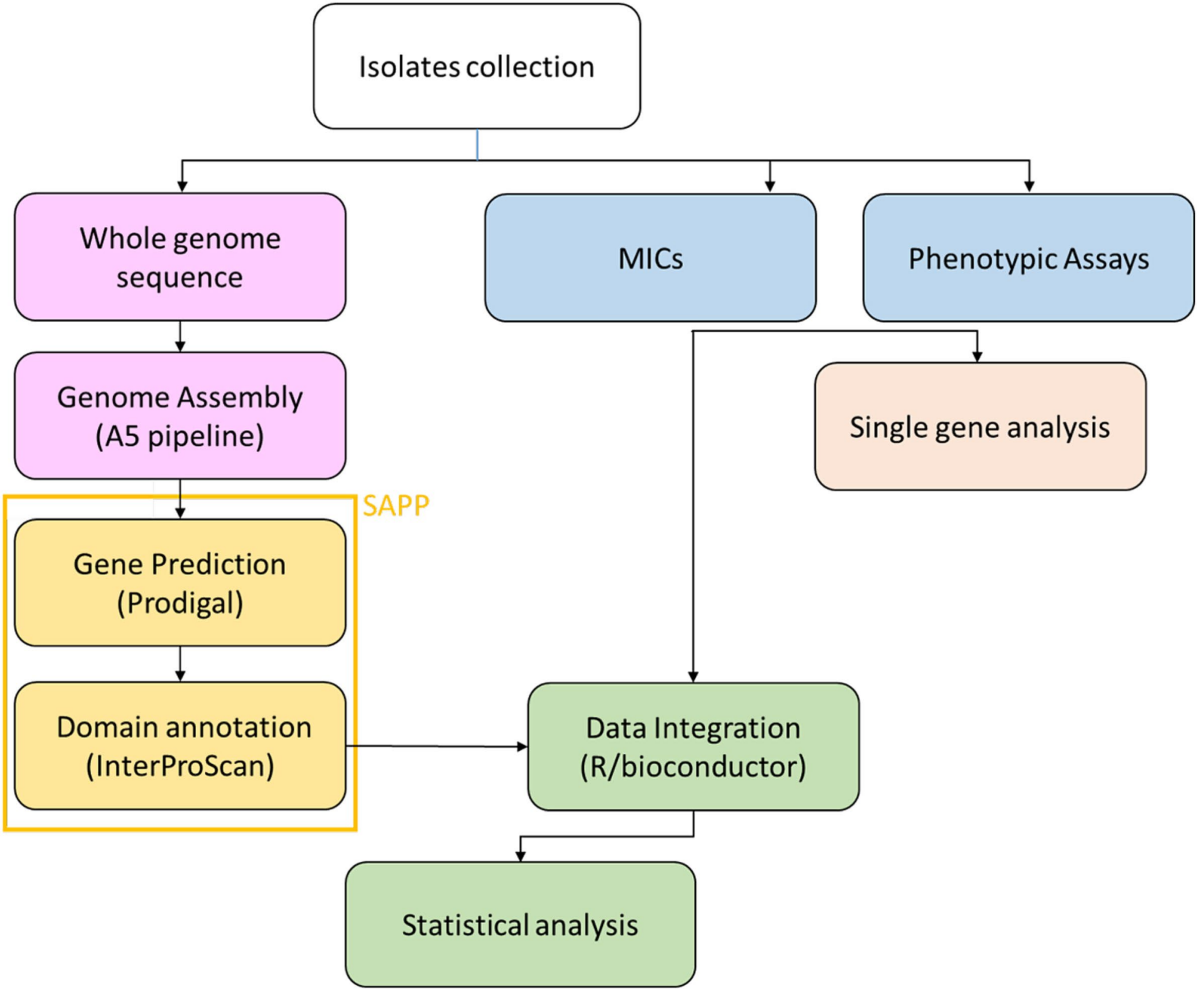
Workflow of the bioinformatics analysis on the clonal strain collection.

Subsequently, the reads were assembled using the A5 pipeline (Coil et al., 2015) and the resulting contigs were imported into the semantic annotation platform SAPP (Koehorst et al., 2018). Protein annotation and domain identification was performed using Prodigal (Hyatt et al., 2010) and InterProScan (Blum et al., 2021), respectively. SNPs calling was done by mapping the raw reads on the genome of the reference strain PAO1 using the software Snippy (Seemann, 2015). The analysis pipeline is represented in Figure 1.

### 2.5 Detection of phylogenetic signal and molecular evolution analysis

The association of phenotypes (encoded as binary traits) with the phylogenetic tree was measured using the function phylo.d, from the R package caper (Fritz and Purvis, 2010). The phylogenetic signal was calculated using two null models: (i) phylogenetic randomness; and (ii) Brownian model.

### 2.6 Data cleaning

The domain matrix containing information on the domain count for each isolate had been cleaned removing uninformative samples and features. A filter based on the number of zeros was applied for each sample and domain. Percentiles were computed for each distribution; the cutoff for the samples was set to the 95th percentile. The cutoff for the features was set to the 80^th^ percentile. Percentiles were chosen to remove as more uninformative features as possible while deleting as few samples as possible to avoid overfitting. Samples and features with near-zero variance were removed from the dataset using “nearZeroVar” from the caret library.

### 2.7 Visualisation techniques

After data cleaning, we proceeded to the exploratory analysis of the domain matrix. To check if there was a natural separation of the three classes between the samples PCA and t-SNE were used. T-SNE were performed using ‘Rtsne‘package (Roberts, 2010) with perplexity = 50, max iteration = 5,000, theta = 0, only two dimensions were computed, while PCA was computed using ‘prcomp‘function, present in the standard ‘stats‘package in R, selecting only the first two components. NMF was used to cluster data in an unsupervised fashion setting k = 2 and 100 iterations.

### 2.8 Feature selection using SAPP

In order to find the possible associations between the variations in domain abundance and the phenotypes/antibiotic resistance, we performed a statistical analysis using the Wilcoxon-tests for each combination and filtered the results applying statistical parameters (*p* < 0.05, Z-score >0.1 in at least one strain).

### 2.9 Statistical analysis of PFAM–phenotype associations

Let X be the matrix of PFAM family counts (families × strains) and Y the matrix of phenotypes (strains × phenotypes). For each family *i* and phenotype *j*, we fit the linear model fit <− lm(X[i,] ~ Y[, j]) and extracted:

1. *β* = coefficient of the phenotype term,
2. 95% confidence interval for β via confint(fit, level = 0.95), and.
3. raw *p*-value for the phenotype effect from anova(fit)[1, “Pr(>F)”].

To control the false discovery rate within each phenotype, raw *p*-values were adjusted using Benjamini–Hochberg [p.adjust(method = “fdr”)], and associations with FDR < 0.10 were deemed significant. Volcano plots (β̂ vs. –log₁₀FDR) were generated with ggplot2 to display effect magnitude against statistical strength.

## 3 Results

### 3.1 Phenotypic characterisation

Each isolate was subjected to testing for 14 distinct phenotypes that are typically lost or gained during the microevolution of *P. aeruginosa* in the airways of patients with cystic fibrosis. In order to identify positive and negative strains for pyocyanin secretion and biofilm formation, a cutoff value was fixed at 0.02 (OD_695_/OD_600_) and at 0.2 (OD_550_/OD_600_), respectively. The low boundaries were set at the results obtained for the strain PAO1, which served as the positive control. With regard to the coloured colony phenotype, it was possible to distinguish between higher (value of 2) and lower (value of 1) pigmentation. Two levels of siderophore secretion were established based on the presence of a more (value of 2) or less (value of 1) marked halo on the plates. The absence of the phenotype is indicated by the value 0. Multidrug-resistant isolates are indicated by the value 1, and intermediate resistant isolates are indicated by the value 0.5. The number and percentage of isolates exhibiting a given phenotype were as follows: ability to lyse erythrocytes (82%); autolytic activity (70%); multidrug resistance (60%); hypermutability (57%); siderophore secretion (35%); pyocyanin secretion. The remaining phenotypes were as follows: biofilm production (13, 32.5%), pyoverdine secretion (12, 30%), pigmented colonies (12, 30%), mucoid phenotype (12, 30%), swarming motility (8, 20%), LasR mutants (6, 19.35%), and protease production (4, 10%). Additionally, an *in-silico* O-antigen serotyping was conducted, which revealed that all the isolates belong to serotype O6. Spearman’s correlation coefficient was employed to ascertain the correlation between each phenotype and the time of isolation, as well as between each pair of phenotypes. In the majority of cases, the correlation coefficient rho was less than 0.5, indicating a weak correlation. However, MDR and hypermutable phenotypes exhibited a correlation value greater than 0.5, while siderophore secretion demonstrated a strong negative correlation with the time of isolation (rho = −0.7, Table 2).

**Table 2:** Spearman’s correlation analysis for each phenotypic assay and time of isolations.

Phenotypic assay	Rho	*p*-value
MDR	0.562	0
Coloured colonies	−0.274	0.088
Mucoidy	−0.407	0.009
Swarming	−0.272	0.09
Twitching	/	/
Haemolysis	−0.288	0.072
Autolysis	0.028	0.862
Siderophore secretion	−0.793	1.04E-09
Pyocyanine secretion	−0.227	0.159
Pyoverdine secretion	−0.442	0.004
Protease secretion	−0.251	0.118
Biofilm	0.2024	0.211
LasR mutants	0.384	0.033
Hypermutability	0.5	0.001

Minimum Inhibitory Concentration (MIC) data for the 40 isolates were previously reported in Bianconi et al. (2019), including values for 12 antibiotics across nine classes. These data are not replicated here to avoid redundancy.

### 3.2 Phylogenetic signal analysis

**Figure 2:**
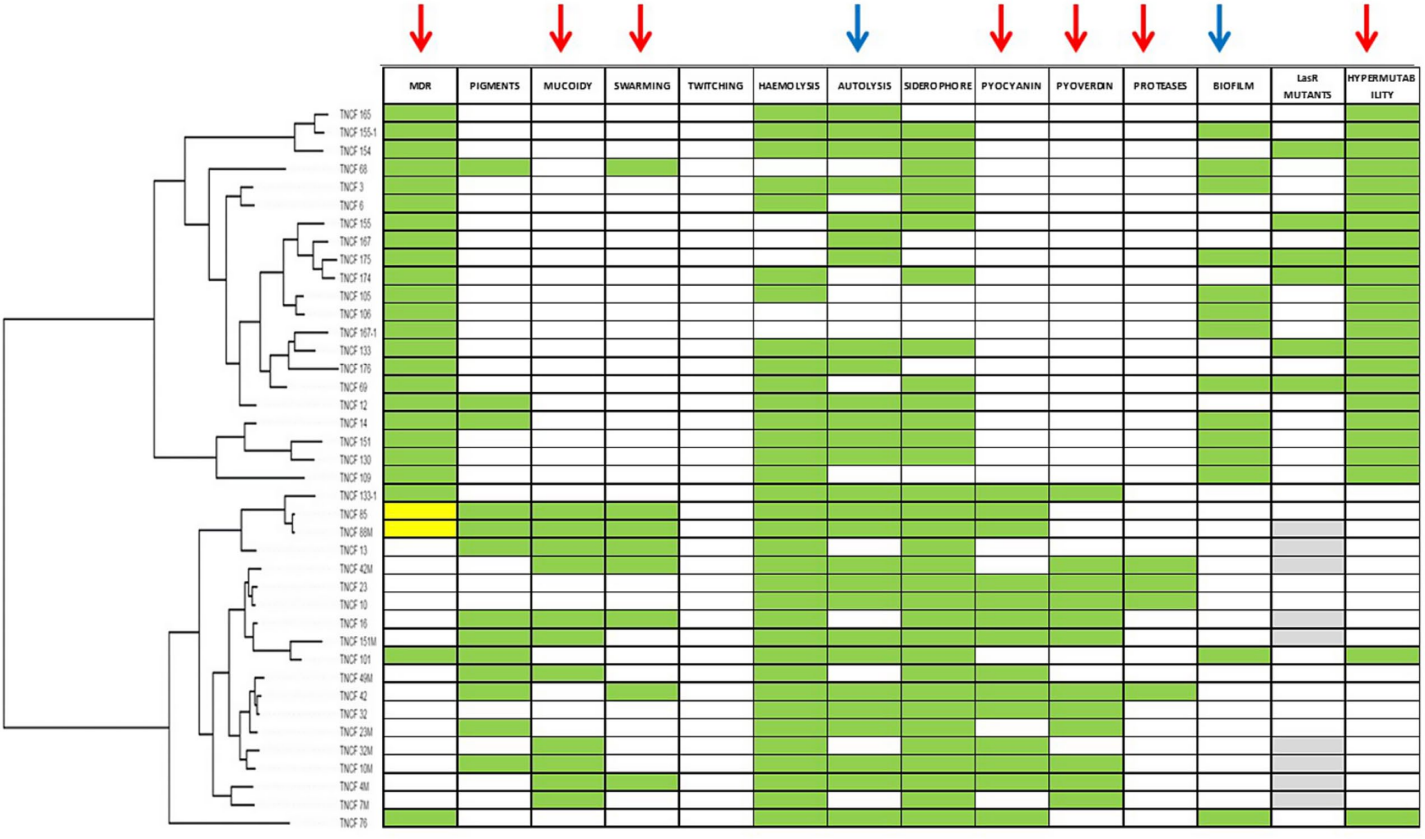
Distribution of phenotypes according to population structure. Green cells indicate positive phenotypes; white cells indicate negative phenotypes; for MDR phenotype yellow cells indicates intermediate resistant isolates; for LasR mutants phenotype grey cells indicates isolates not tested. Arrows indicate phenotypes for which Phylo.d produced a significant output. Red arrows, phylogenetic signal according to Brownian model; blue arrows, random (negative) phylogenetic signal.

To verify if the presence of a phenotype was associated to the phylogenetic tree (constructed based on core genome SNPs) we performed an analysis of phylogenetic signal (Figure 2). Binary results of each phenotypic assay (encoded as 0 for absence and 1 for presence of the phenotype), were tested with the phylo.d function of R. Table 3 reports the results of the analysis with the estimated D, relative to the phylogenetic signal, the *p*-value for D ≠ 1 and the p-value for D ≠ 0. Seven phenotypes (MDR, mucoid, swarming motility, pyocyanin, pyoverdine and protease secretion, and hypermutability) were distributed on the tree according to the Brownian model, indicating that these traits are significantly phylogenetically clumped and not randomly distributed as confirmed by the significant p-value (< 0.05) for D ≠ 1; autolysis and biofilm production traits are distributed according to a random model, which indicates an over dispersed distribution of these traits (the estimated D close to 1, typical of a random distribution of the characters across the phylogenetic tree), while for the remaining phenotypes the distribution model could not be determined (Figure 2). For two phenotypes it was not possible to perform the phylogenetic signal analysis: the twitching motility trait had a single trait (negative for all isolates) and the LasR mutant trait was tested for only 31 isolates.

**Table 3:** Phylo.d analysis for each phenotypic assay.

Phenotypic assay	Estimated D	*p*-value (D ≠ 1)	*p*-value (D ≠ 0)
MDR	−0.419	0	0.881
Coloured colonies	0.581	0.069	0.057
Mucoidy	−0.106	0	0.631
Swarming	0.225	0.024	0.339
Twitching	/	/	/
Haemolysis	0.491	0.129	0.201
Autolysis	1.12	0.639	0
Siderophore secretion	0.692	0.208	0.076
Pyocyanine secretion	−0.248	0	0.75
Pyoverdine secretion	−0.347	0	0.81
Protease secretion	−0.16	0.048	0.552
Biofilm	0.696	0.124	0.038
LasR mutants	/	/	/
Hypermutability	−0.539	0	0.925

### 3.3 Genotypic characterisation

The genome sequences of the 40 strains were used to study the genotypic-phenotypic correlation of the isolates. Candidate genes were extrapolated from the virulence factors database VFDB.

Six genes encoding flagellar proteins were deleted in all isolates: *flgK, flgL, fliC, flaG, fliD and fliS*. Other genes showed frameshift mutations or premature stop codon, however, no one of the alterations found seems to be associated with the loss of swarming motility phenotypes. Although flagellar proteins are not directly responsible for antimicrobial resistance, their deletion is often associated with a shift to non-motile phenotypes and increased biofilm formation. This transition enhances persistence and tolerance, indirectly contributing to reduced antibiotic susceptibility (Wei et al., 2025). We did not found *pilA, pilB, pilC and pilQ* genes, involved in type IV pili biosynthesis. Genes involved in the mismatch repair system, siderophore, phenazines biosynthesis, and quorum sensing did not present any mutation able to explain the presence or absence of the phenotype.

In our previous study (Bianconi et al., 2019), we analysed the mutations present in genes involved in antibiotic resistance, alginate production, and biofilm formation. Variations present in the MDR isolates but not in the susceptible ones were found in *gyrB*, *parC* and *parE* (involved in resistance to fluoroquinolones), *pmrB* (involved in resistance to polymyxin) and *oprD* (involved in the resistance to carbapenems).

### 3.4 Variations in functional domains

**Figure 3:**
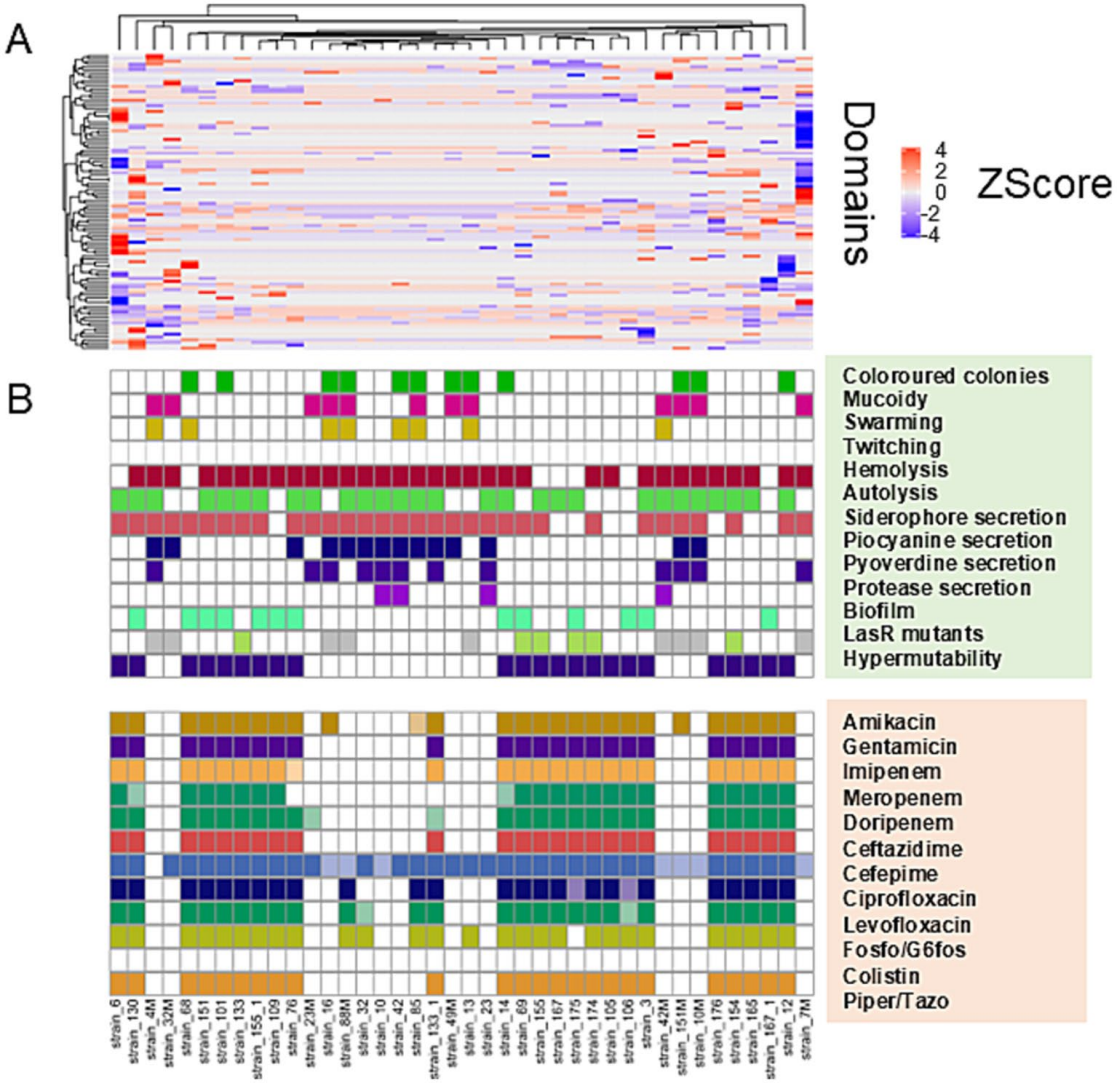
Hierarchical clustering/heatmap PFAM domains. Hierarchical clustering was performed on the PFAM data matrix Z-score of the functional domains (*n* = 2,791) and visualised as a heatmap **(A)**. Individual phenotypes or antibiotics resistance class of each genome\strain are represented in the panel **B**.

**Figure 4:**
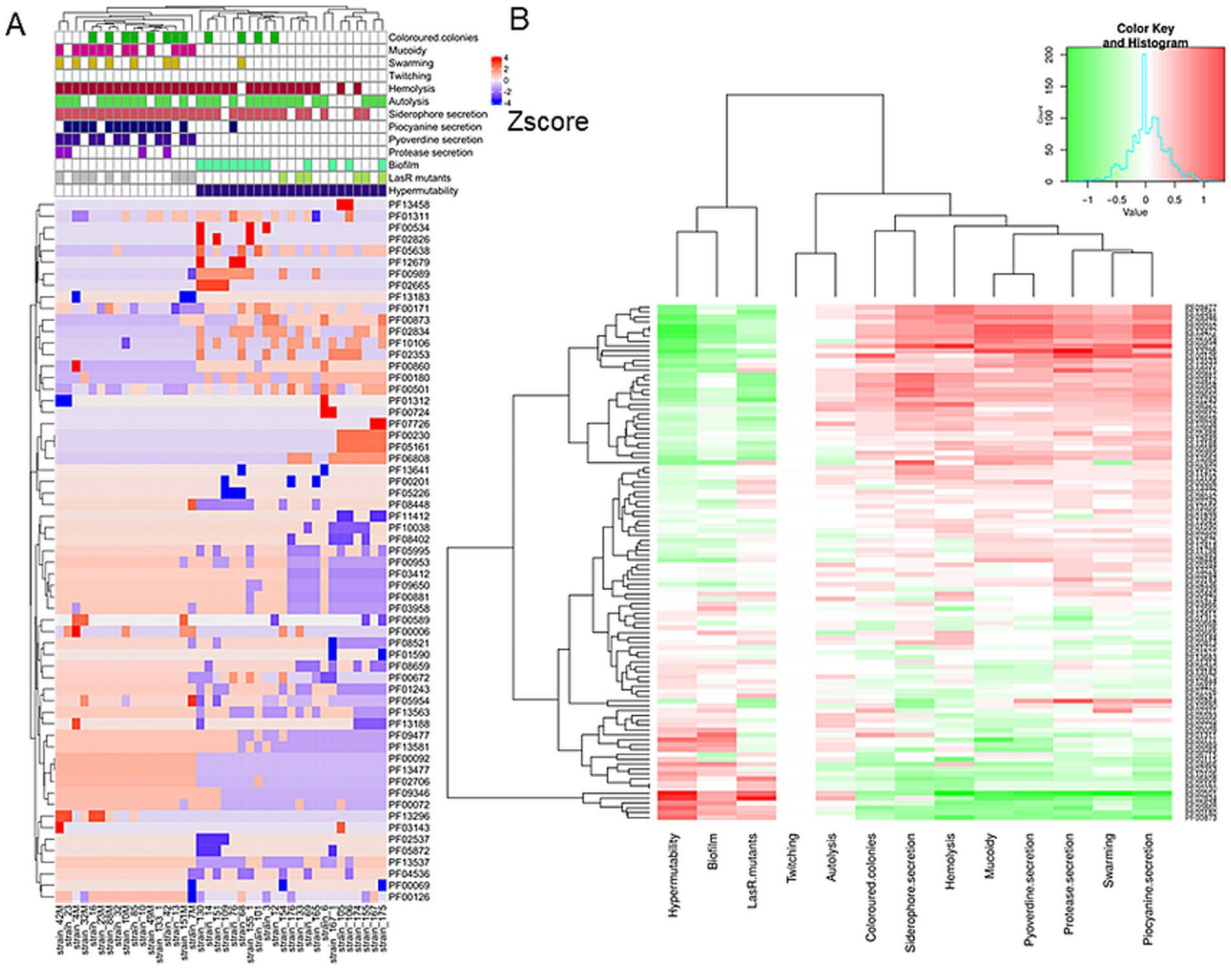
Hierarchical clustering and heatmap of PFAM domains. Hierarchical clustering was performed on the PFAM data matrix Z-score and visualised as a heatmap **(A)**. The panel **B** shows the heatmap of the differential mean Z-score of the domains between the subgroups (presence/absence) of each phenotype.

**Figure 5:**
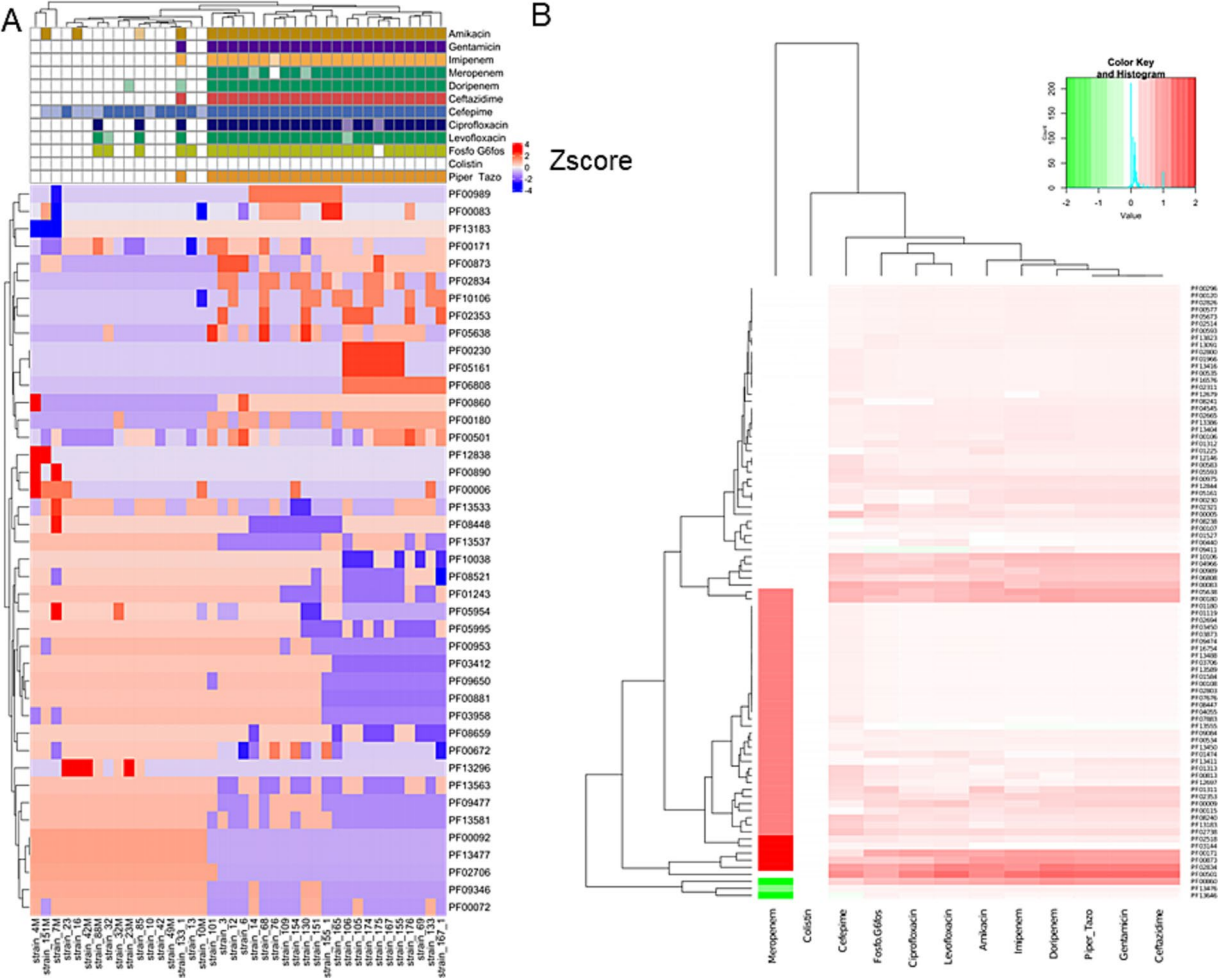
Hierarchical clustering and heatmap of PFAM domains. Hierarchical clustering was performed on the PFAM data matrix Z-score and visualised as a heatmap **(A)**. The panel **B** shows the heatmap of the differential mean Z-score of the domains between the subgroups (presence/absence) of each antibiotic resistance.

Hierarchical clustering was performed on the PFAM data matrix of the functional domains and visualised as a heatmap (Figure 3), in which the individual phenotypes of each genome/strain is represented in the bottom panels in the figure. We found a total of 2,791 domains, but most of them did not show variations with respect to the presence/absence of the phenotype sought; therefore, from this analysis, it was not possible to observe the presence of PFAM domains pattern associated with the phenotypes tested. However, the clusters formed by the strains (the columns in Figure 3), were consistent with the patterns formed by the Z-scores. This group of strains featured common phenotypic patterns, namely susceptibility to antibiotics and secretion of pyocyanin and pyoverdine. Such phenotypes are typical of the isolates from acute infection. On the other hand, the group of MDR strains presented a number of domains which had larger absolute values of Z-score. We found a total of 276 domains with a statistically significant different abundance in strains with or without the phenotypes or antibiotic resistance class for specific antibiotics and, most notably, this analysis grouped different phenotypes by their association with the functional domains (Figure 4). This was clearer for the antibiotic resistance, which showed a distinct pattern of domain abundance in resistant strains (Figure 5). In fact, we were able to identify 87 domains associated with the phenotypes not connected to antibiotics and 189 functional domains that were significantly enriched in the resistant strains compared to the susceptible ones (Figures 4, 5, respectively, and Table 4). Several domains related to antibiotic resistance were found in dehydrogenases and oxidases enzymes, but we also found enrichment in functional domains belonging to proteins commonly associated with antibiotic resistance, such as outer membrane proteins, outer membrane porins and efflux pumps; the same domains are shared with many hypothetical proteins with unknown function.

**Table 4:** Functional domains with different abundance in resistant strains respect to susceptible ones.

PHP domain	Aminoglycosides	Carbapenems	Cephalosporines	Fluoroquinolones	Phosphonic acids	Penicillins
PF00005	0.2083	0.1768	0.2083	0.2267	0.2707	0.2083
PF00009	0.375	0.1818	0.375	0.4267	0.3191	0.375
PF00083	0.5208	0.4293	0.5208	0.52	0.5271	0.5208
PF00115	0.25	0.298	0.25	0.2267	0.1795	0.25
PF00171	0.7083	0.7727	0.7083	0.68	0.6296	0.7083
PF00180	0.6458	0.6162	0.6458	0.5067	0.4387	0.6458
PF00230	0.25	0.2727	0.25	0.1333	0.1083	0.25
PF00501	1.1667	1.1212	1.1667	1.1467	0.6667	1.1667
PF00586	0.7708	0.7778	0.7708	0.6133	0.5071	0.7708
PF00873	0.75	0.8434	0.75	0	0.396	0.75
PF00989	0.4375	0.3636	0.4375	0.4267	0.4103	0.4375
PF01311	0.375	0.1313	0.375	0.1333	0.4046	0.375
PF02321	0.1857	0.0909	0.1857	0.24	0.359	0.1857
PF02353	0.3333	0.3636	0.3333	0.2133	0.2963	0.3333
PF02738	0.2708	0.2576	0.2708	0.28	0.3048	0.2708
PF02834	1	0.8889	1	0.96	0.661	1
PF04966	0.4792	0.5101	0.4792	0.4667	0.3333	0.4792
PF05161	0.25	0.2727	0.25	0.1333	0.1083	0.25
PF05638	0.6458	0.7172	0.6458	0.5067	0.5027	0.6458
PF08240	0.2292	0.1869	0.2292	0.2533	0.3105	0.2292
PF10106	0.5208	0.5556	0.5208	0.4	0.3704	0.5208

**Figure 6:**
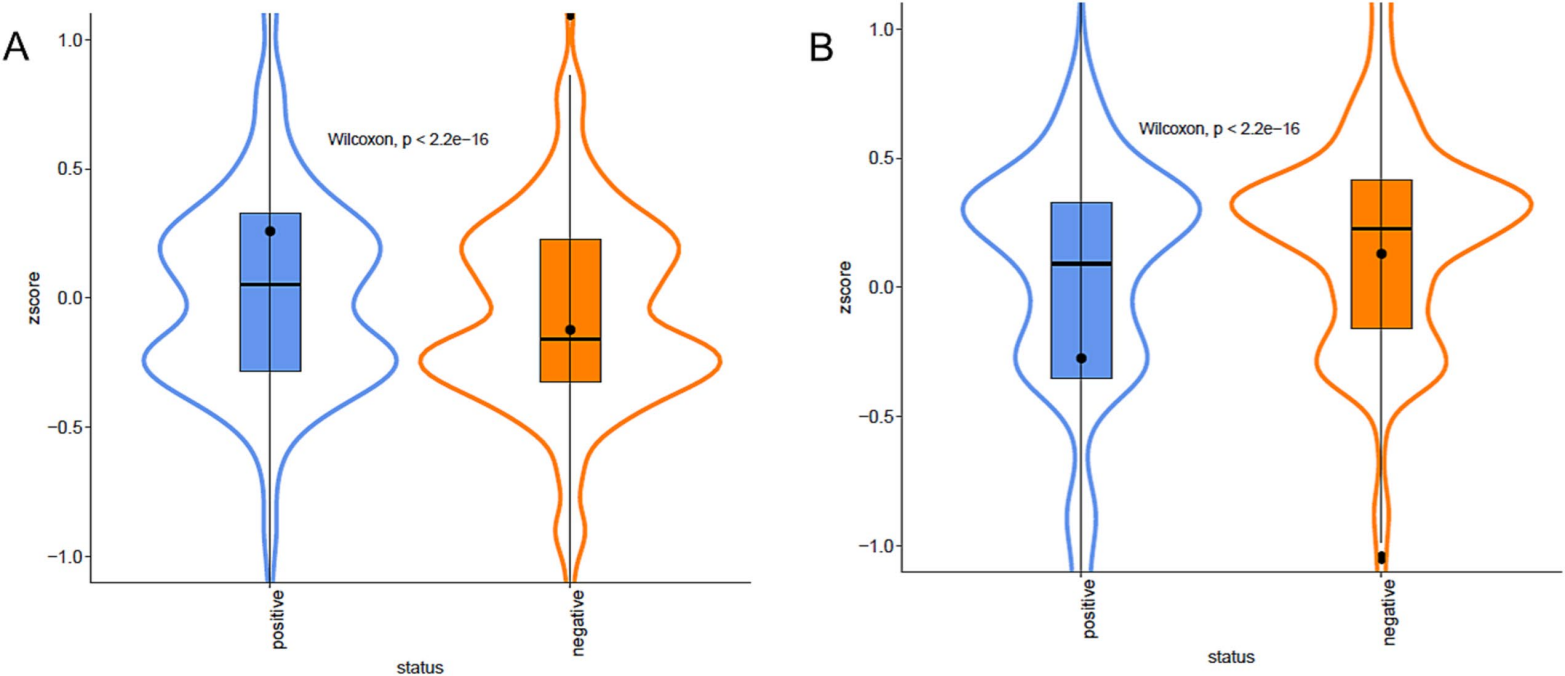
Violin plots of Biofilm formation. Violin plots of the Z-score of the domains associated, positive **(A)** or negative **(B)**, with biofilm formation capacity, grouped by presence/absence categories. The colour represents the different categories. Data are described by boxplot and Z-score mean (black circle).

**Figure 7:**
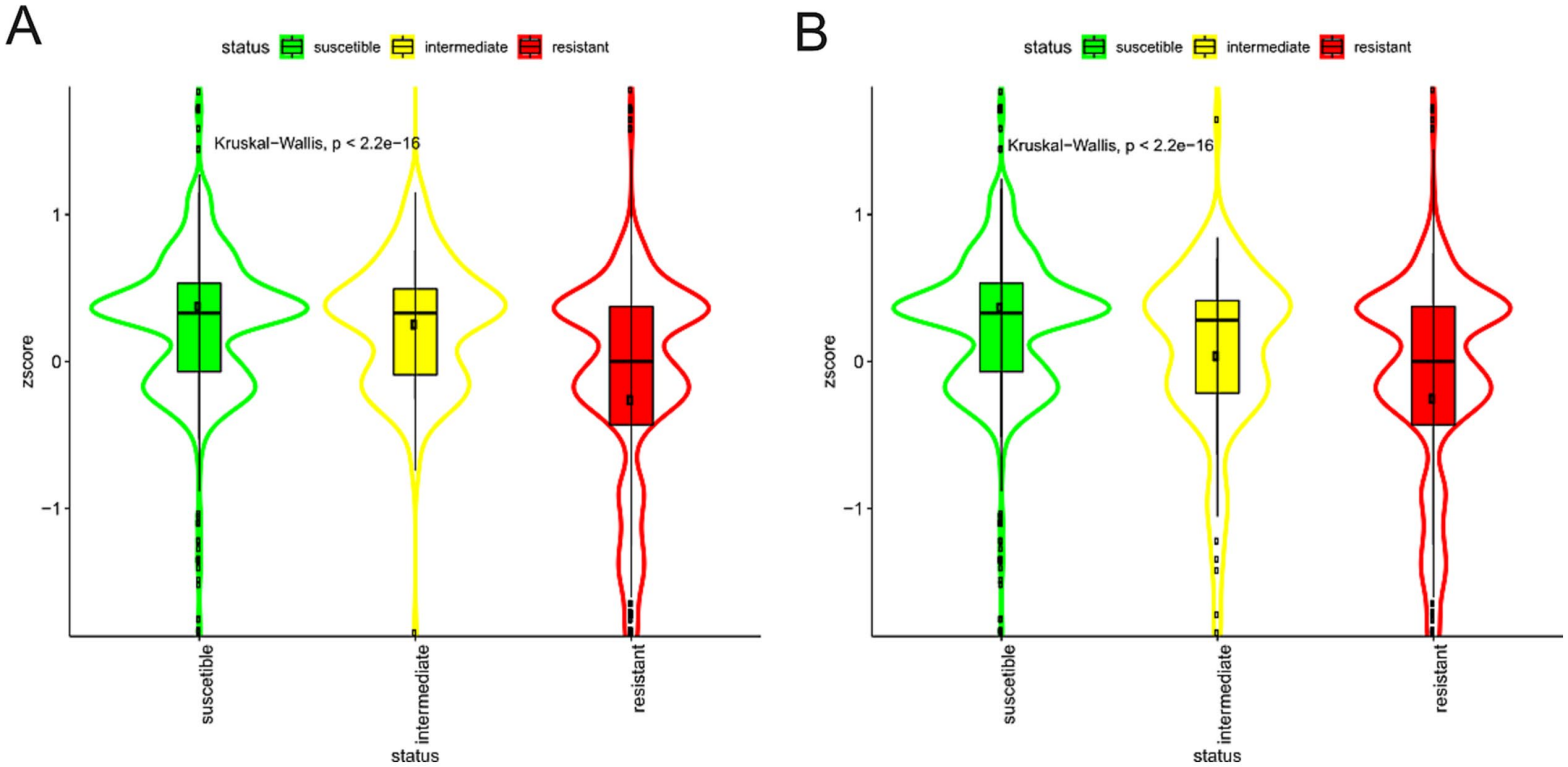
Violin plots of Imipenem resistance. Violin plots of the Z-score of the domains associated, positive **(A)** or negative **(B)**, with Imipenem resistance, grouped by presence/absence categories. The colour represents the different categories. Data are described by boxplot and Z-score mean (black circle).

**Figure 8:**
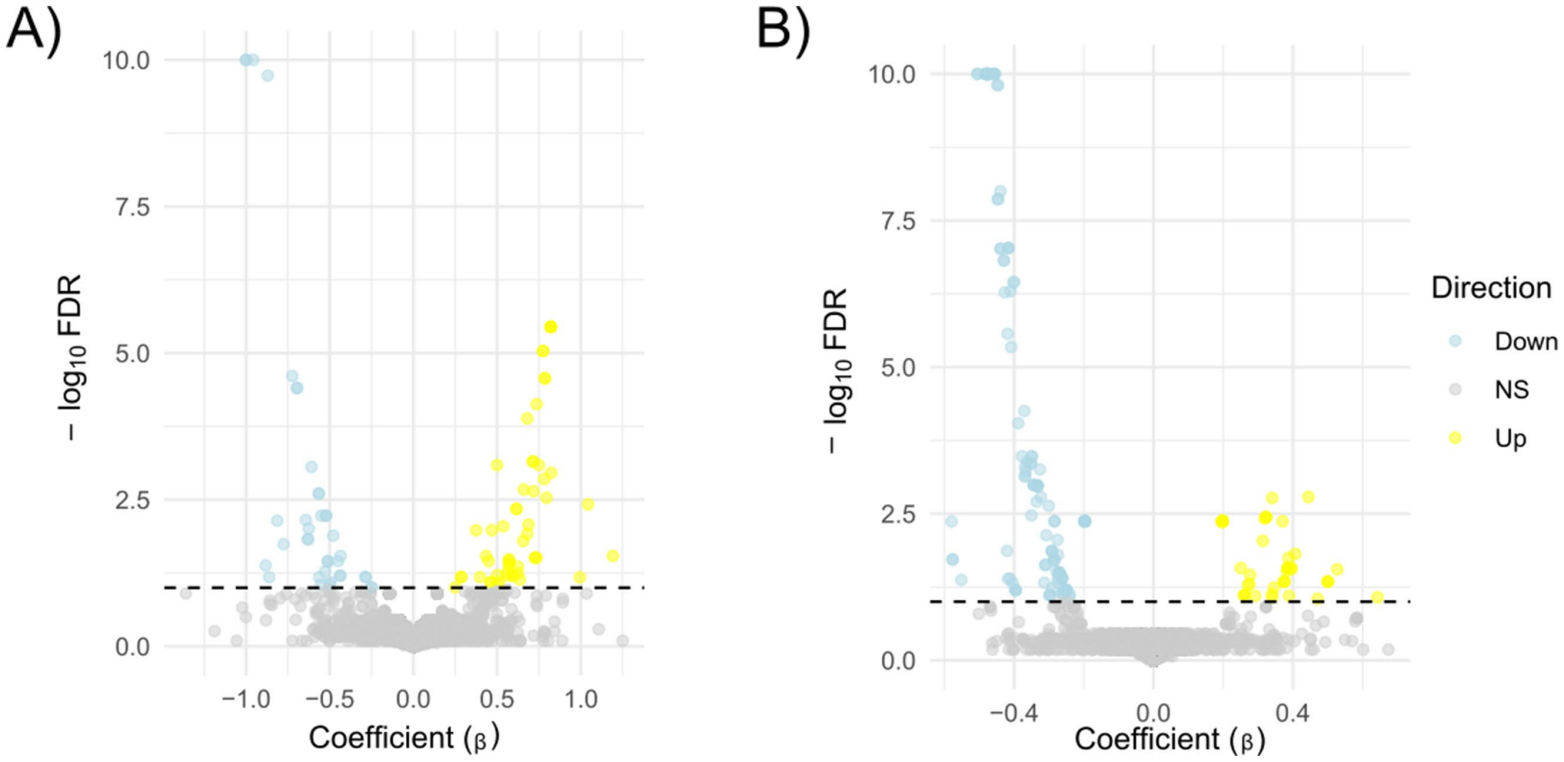
Volcano plots of PFAM–phenotype associations. Each point represents one PFAM family tested against a single phenotype (panel **A**) or resistance to an antibiotic (panel **B**). The x-axis shows the estimated effect size (*β*, from linear regression of PFAM abundance on phenotype), and the y-axis shows –log₁₀(FDR), where FDR is the Benjamini–Hochberg–adjusted *p*-value for that phenotype (capped at 10 for display). The horizontal dashed line marks the significance threshold (FDR = 0.10, −log₁₀(FDR) = 1). Points coloured yellow are significant with β > 0 (“Up”), light blue are significant with β < 0 (“Down”), and grey are non-significant.

We then split the domains into two groups: (i) the UP group composed of the domains more abundant in the strains associated with the presence of a phenotype and (ii) the DOWN group composed by the domains more abundant in the strains characterised by the absence of the phenotype. For each case, we plot the violin plots of each strain separately (not shown) or grouped together. Lastly, we test if the Z-score differences of the domain’ groups were statistically significant (Wilcoxon test or Kruskal-Wallis test for phenotypes and resistance/susceptibility, respectively). We found a robust and significantly different abundance (enrichment or depletion) in domains related to several phenotypes and antibiotic resistance, most interesting among them; we found a significant differential abundance in domains associated with biofilm formation, mucoid phenotype and resistance to Carbapenems (Figures 6, 7). To ensure control of false positives and to convey both the magnitude and confidence of each association, we re-analysed all PFAM-phenotype pairs using linear regression. For each family×phenotype combination, we fit a linear model in R (lm), extracted the regression coefficient (*β*) and its 95% confidence interval, and obtained a raw *p*-value from the ANOVA table. We then applied the Benjamini–Hochberg procedure separately to each phenotype’s p-value vector (FDR < 0.10). Significant associations are those with per-phenotype FDR < 0.10. In particular we obtain 41 and 53 differential domains for the two classes of phenotypes. To visualise both effect size and adjusted significance, we introduce a new volcano-plot panel (Figure 8), plotting β against –log₁₀(FDR). Full results—β, confidence bounds, raw p, and FDR for every PFAM family and phenotype—are provided in Supplementary Table 1.

## 4 Discussion

*Pseudomonas aeruginosa* is the most common airway pathogen in CF patients (Gibson et al., 2003; Blanchard and Waters, 2019). Its extensive repertoire of regulatory genes facilitates adaptation to the CF lung environment through both genotypic and phenotypic modifications, resulting in a broad spectrum of morphotypes (Folkesson et al., 2012; Iwańska et al., 2023; Weimann et al., 2024). These adaptations include pathoadaptive mutations and horizontal gene acquisition, which contribute to the emergence of multidrug-resistant (MDR) strains and complicate therapeutic management (Weimann et al., 2024).

A deeper understanding of the genotypic and phenotypic changes that *P. aeruginosa* undergoes during adaptation is fundamental to identify novel therapeutic approaches (Mayer-Hamblett et al., 2014; Sousa and Pereira, 2014; Camus et al., 2021; Rossi et al., 2021). Building on our previous findings (Bianconi et al., 2019), which found limited correlation between strain-specific mutations and phenotypic traits, we adopted a novel bioinformatic approach to explore associations between broader genetic features and phenotypic adaptations.

In CF infections, *P. aeruginosa* progressively loses most of the virulence factors and acquires phenotypes able to confer antibiotic resistance over time (Winstanley et al., 2016). In our isolate collection, pigmentation, swarming motility, and siderophore production were predominantly observed in early and intermediate isolates. Alginate and pyoverdine production were largely confined to early isolates, while protease secretion was exclusive to early-stage strains. Conversely, MDR and hypermutable phenotypes were primarily associated with late-stage isolates, and LasR mutants were also more prevalent in the later stages of infection.

Phenotypic traits showed clear temporal patterns across the longitudinal isolates. Traits associated with acute infection—such as pigmentation, siderophore secretion, motility, and protease activity—were predominantly found in early-stage isolates (Bianconi et al., 2019), conversely, traits linked to chronic adaptation, such as multidrug resistance and hypermutability, were more common in later-stage isolates. Spearman’s analysis performed to study the correlation of the phenotypes showed an expected strong correlation between hypermutability and MDR phenotypes (all the hypermutable isolates are also resistant to most of the antibiotics tested). A hypermutable phenotype leads to the accumulation of mutations that can confer antibiotic resistance to the bacterium, as well as an enhanced genetic adaptation to CF airway (Hogardt et al., 2007; Feliziani et al., 2014). Not surprisingly, pyoverdine (a pigmented siderophore involved in iron uptake) correlated positively with pyocyanin (which is also involved in the reduction of iron allowing its uptake; Cornelis and Dingemans, 2013). We found a negative correlation between pyoverdine secretion and MDR and a positive correlation between biofilm formation and hypermutability, probably due to the adaptive evolution of *P. aeruginosa* leading to the typical phenotypes of strains from chronic infections (Marvig et al., 2013).

The analysis of the mutations in the genes recognised to be involved in the phenotypes, included MDR, was partially performed in our previous study (Bianconi et al., 2019). Here we completed the analysis for all the phenotypes tested in this study. Briefly, different genes involved in bacterial motility were absent in our isolates, such as *fliC, pilB and pilQ* that are reported to be mutated in non-motile strains, with a premature stop codon or frameshift mutations. No one of these mutations is able to explain the motile or non-motile phenotype of our *P. aeruginosa* population. The same situation is shown for genes involved in the production of virulence factors; for instance, none of the 40 isolates presented the specific mutations found in the mismatch repair genes leading to a hypermutable phenotype. We confirmed our previous findings, in line with other studies (Pommerenke et al., 2010; Klockgether et al., 2013; Jeukens et al., 2014; Rojas et al., 2022), even if we found several gene deletions, frameshift mutations and SNPs, however, under no circumstance, it was possible to determine a precise correlation between genotype and phenotype of the isolates. It is important to note that a specific phenotype is often the result of the interaction between multiple gene products (Jochumsen et al., 2016). Consequently, establishing a correlation between phenotype and genotype by analysing single-gene mutations can be challenging. Additionally, it is possible that mutations in genes not directly associated with the phenotype or yet to be characterised may also contribute to the presence or absence of the phenotype.

Our results support several key conclusions: (i) virulence factors associated with acute infection are progressively lost, while traits characteristic of chronic colonisation are acquired; (ii) the microevolutionary patterns observed in our longitudinal collection are consistent with established evolutionary trajectories; and (iii) although signatures of pathoadaptive mutations were detected, their direct association with phenotypic traits remains elusive.

Despite the study being limited to a single patient, the longitudinal design provides a high-resolution perspective on within-host evolution in a stable genetic background, minimising inter-host variability. This approach has proven valuable in previous work (Bianconi et al., 2019) and is supported by recent studies (Thöming and Häussler, 2022; Martínez-Gallardo et al., 2024), which highlight the clinical relevance of within-host evolutionary trajectories in *P. aeruginosa*.

To further explore the possible association between genetic features and phenotypes, we used SAPP, a bioinformatics tool for comparative functional genomics (Koehorst et al., 2018), to examine how specific protein functional domains were enriched or depleted across the strain population. The screening of protein domains rather than full-length proteins overcomes the limits due to the high number of hypothetical proteins with unknown function and poorly annotated proteins due to wrong homology inference (Richardson and Watson, 2013; Vincent, 2024). Furthermore, using the protein domains adds a new level of genomic exploration, allowing a more accurate annotation of proteins and feasibly discovering new functions of known proteins (Lobb et al., 2020). We acknowledge that SAPP detects domain-level variation but does not capture amino acid substitutions within conserved domains. Since even single mutations can drastically alter domain function, this limitation may result in an incomplete characterisation of functional diversity. Bacteria living within biofilms are isolated and able to survive even high-dose antibiotic treatments, and therapeutic strategies targeting the mechanisms underlying biofilm-associated antibiotic resistance are currently under development (Ciofu and Tolker-Nielsen, 2019). It is therefore crucial to understand the differences in functional domains related to the biofilm formation. Our domain-based analysis revealed that specific protein domains, such as PAS domain *PF00989*, were significantly associated with biofilm formation. PAS domains are known to mediate environmental sensing and have been implicated in biofilm regulation in other bacteria.

Another domain, namely *PF08448*, which was underrepresented in the biofilm producing strain is also annotated as a PAS fold domain, involved in signalling and binding to gaseous compounds (Hefti et al., 2004; Yu et al., 2023). To our knowledge, the involvement of those two domains in biofilm formation has not been directly investigated in other studies although their presence is reported in functional studies on the regulation of biofilm formation in *P. aeruginosa* and *Xanthomonas campestris* (Mikkelsen et al., 2013; Hsiao et al., 2011).

We also identified domains related to CdrA adhesins, which contribute to the structural integrity of the biofilm matrix (Reichhardt, 2023).

The variations in protein functional domains between susceptible and resistant strains revealed a highly significant association between the enrichment or depletion of specific domains (some of them with uncharacterised functions) and the associated phenotypic traits.

We were able to single out 87 domains related to phenotypic adaptation not connected to antibiotic resistance and 189 functional domains that were significantly enriched in the antibiotic-resistant strains compared to the susceptible ones; we found domains commonly associated with antibiotic resistances, which were significantly enriched or depleted in the resistant strains. For examples domains associated with outer membrane efflux pumps and porins, as well as a number of domains with a still unknown function. It is well known that efflux pumps are crucial for bacterial resistance to several antibiotics and they are attractive targets for antibacterial drugs (Lamut et al., 2019; e.g., broad-spectrum efflux pumps inhibitors have been shown to potentiate of the action fluoroquinolones, levofloxacin; Mahmood H. et al., 2016); on the other hand porins deficiency (either by loss of expression of porin-encoding genes or mutations) has been described as a leading mechanism to the emergence of Carbapenem-resistant *P. aeruginosa* (Nordmann and Poirel, 2019; Dulanto Chiang and Dekker, 2024). Our data suggests that the domains with an unknown function may also be associated with antibiotic resistance mechanisms, although the description of the molecular basis of these mechanisms is still challenging. Consequently, further in-depth study and characterisation of these domains could lead to the identification of new targets for the development of molecules with antimicrobial functions or adjuvants to antibiotics already used in clinical practice.

Our findings are further supported by recent comparative genomic studies. Sahayarayan et al. (2024) identified resistance-associated genes in *P. aeruginosa*, including those involved in *β*-lactam resistance, efflux regulation, and outer membrane permeability, which align with our observation of enriched domains related to efflux pumps and porins. Similarly, Ahmed (2022) demonstrated that multidrug-resistant strains harbour a broader spectrum of resistance genes, with a direct correlation between gene count and phenotypic resistance. This is consistent with our domain-based analysis, where MDR isolates exhibited a higher abundance of domains linked to aminoglycoside resistance, β-lactamase activity, and efflux transport.

In our collection of clonal isolates there are significant differences in the functional domains abundance in the MDR isolates with respect to the susceptible ones. However, it has not yet been possible to determine which specific feature is the most suitable for the design of new molecules active against the associated phenotype. This was out the focus of this study, but it will be the focus of subsequent studies.

In conclusion, our integrative approach combining phenotypic profiling with domain-level comparative genomics revealed consistent associations between conserved functional domains and clinically relevant traits such as biofilm formation and multidrug resistance. While gene-centric approaches catalogue known resistance determinants, our domain-level analysis captures the functional architecture underlying these genes, including those in hypothetical or poorly annotated proteins. Together, these studies reinforce the multifactorial nature of resistance in *P. aeruginosa* and validate the utility of domain-based comparative genomics in identifying conserved molecular features relevant to pathogenesis.

While the results are promising, further analyses are needed to validate these findings. Future research could benefit from applying more advanced techniques, such as Support Vector Machines or Deep Neural Networks, to larger datasets to uncover additional druggable targets.

## Data Availability

The datasets presented in this study can be found in online repositories. The names of the repository/repositories and accession number(s) can be found at: https://www.ncbi.nlm.nih.gov/genbank/, PRJNA326244.

## Supplementary material

SUPPLEMENTARY TABLE 1 A long-format table with one row for every PFAM family × phenotype combination. It summarises both linear‐model and Wilcoxon test results, sample sizes, and PFAM prevalence/variance.

## References

[ref1] Ahmed O. B. (2022). Detection of antibiotic resistance genes in *Pseudomonas aeruginosa* by whole genome sequencing. Infect Drug Resist. 15, 6703–6709. doi: 10.2147/IDR.S389959 36425153 PMC9680685

[ref2] Alexander D. B. Zuberer D. A. (1991). Use of chrome azurol S reagents to evaluate siderophore production by rhizosphere bacteria. Biol. Fertil. Soils 12, 39–45. doi: 10.1007/BF00369386

[ref3] Bhagirath A. Y. Li Y. Somayajula D. Dadashi M. Badr S. Duan K. (2016). Cystic fibrosis lung environment and *Pseudomonas aeruginosa* infection. BMC Pulm. Med. 16:174. doi: 10.1186/s12890-016-0339-5 27919253 PMC5139081

[ref4] Bianconi I. Aschbacher R. Pagani E. (2023). Current uses and future perspectives of genomic technologies in clinical microbiology. Antibiotics 12:1580. doi: 10.3390/antibiotics12111580 37998782 PMC10668849

[ref5] Bianconi I. D’Arcangelo S. Benedet M. Bailey K. E. Esposito A. Piffer E.. (2016). Draft genome sequences of 40 *Pseudomonas aeruginosa* clinical strains isolated from the sputum of a single cystic fibrosis patient over an 8-year period. Genome Announc. 4:e01205-16. doi: 10.1128/genomeA.01205-16 27979930 PMC5159563

[ref6] Bianconi I. D’Arcangelo S. Esposito A. Benedet M. Piffer E. Dinnella G.. (2019). Persistence and microevolution of *pseudomonas aeruginosa* in the cystic fibrosis lung: a single-patient longitudinal genomic study. Front. Microbiol. 9:3242. doi: 10.3389/fmicb.2018.03242 30692969 PMC6340092

[ref7] Bianconi I. Milani A. Cigana C. Paroni M. Levesque R. C. Bertoni G.. (2011). Positive signature-tagged mutagenesis in *Pseudomonas aeruginosa*: tracking patho-adaptive mutations promoting airways chronic infection. PLoS Pathog. 7:e1001270. doi: 10.1371/journal.ppat.1001270 21304889 PMC3033382

[ref8] Bjarnsholt T. Jensen P. Ø. Fiandaca M. J. Pedersen J. Hansen C. R. Andersen C. B.. (2009). *Pseudomonas aeruginosa* biofilms in the respiratory tract of cystic fibrosis patients. Pediatr. Pulmonol. 44, 547–558. doi: 10.1002/ppul.21011 19418571

[ref9] Blanchard A. C. Waters V. J. (2019). Microbiology of cystic fibrosis airway disease. Semin. Respir. Crit. Care Med. 40, 727–736. doi: 10.1055/s-0039-1698464 31887768 PMC7117079

[ref10] Blum M. Chang H. Y. Chuguransky S. Grego T. Kandasaamy S. Mitchell A.. (2021). The InterPro protein families and domains database: 20 years on. Nucleic Acids Res. 49, D344–D354. doi: 10.1093/nar/gkaa977 33156333 PMC7778928

[ref11] Breidenstein E. B. M. de la Fuente-Núñez C. Hancock R. E. W. (2011). *Pseudomonas aeruginosa*: all roads lead to resistance. Trends Microbiol. 19, 419–426. doi: 10.1016/j.tim.2011.04.005 21664819

[ref12] Brown M. R. Foster J. H. (1970). A simple diagnostic milk medium for *Pseudomonas aeruginosa*. J. Clin. Pathol. 23, 172–177. doi: 10.1136/jcp.23.2.172 4987392 PMC474488

[ref13] Butler M. S. Vollmer W. Goodall E. C. A. Capon R. J. Henderson I. R. Blaskovich M. A. T. (2024). A review of antibacterial candidates with new modes of action. ACS Infect Dis. 10, 3440–3474. doi: 10.1021/acsinfecdis.4c00218 39018341 PMC11474978

[ref14] Camus L. Vandenesch F. Moreau K. (2021). From genotype to phenotype: adaptations of *Pseudomonas aeruginosa* to the cystic fibrosis environment. Microb. Genom. 7. doi: 10.1099/mgen.0.000513 PMC8190622 33529147

[ref15] Chan K. G. (2016). Whole-genome sequencing in the prediction of antimicrobial resistance. Expert Rev. Anti-Infect. Ther. 14, 617–619. doi: 10.1080/14787210.2016.1193005 27215476

[ref16] Ciofu O. Tolker-Nielsen T. (2019). Tolerance and resistance of *pseudomonas aeruginosa*biofilms to antimicrobial agents-how *P. aeruginosa*Can escape antibiotics. Front. Microbiol. 10:913. doi: 10.3389/fmicb.2019.00913 31130925 PMC6509751

[ref17] Coil D. Jospin G. Darling A. E. (2015). A5-miseq: An updated pipeline to assemble microbial genomes from Illumina MiSeq data. Bioinformatics 31, 587–589. doi: 10.1093/bioinformatics/btu661 25338718

[ref9001] Cornelis P. Dingemans J. (2013). Pseudomonas aeruginosa adapts its iron uptake strategies in function of the type of infections. Front. Cell. Infect. Microbiol. 3:75. doi: 10.3389/fcimb.2013.00075 24294593 PMC3827675

[ref18] Cramer N. Klockgether J. Tümmler B. (2023). Microevolution of *Pseudomonas aeruginosa* in the airways of people with cystic fibrosis. Curr. Opin. Immunol. 83:102328. doi: 10.1016/j.coi.2023.102328 37116385

[ref19] Davies D. G. Parsek M. R. Pearson J. P. Iglewski B. H. Costerton J. W. Greenberg E. P. (1998). The involvement of cell-to-cell signals in the development of a bacterial biofilm. Science 280, 295–298. 9535661 10.1126/science.280.5361.295

[ref20] Dulanto Chiang A. Dekker J. P. (2024). Efflux pump-mediated resistance to new beta lactam antibiotics in multidrug-resistant gram-negative bacteria. Commun. Med. 4:170. doi: 10.1038/s43856-024-00591-y 39210044 PMC11362173

[ref21] Elfadadny A. Ragab R. F. AlHarbi M. Badshah F. Ibáñez-Arancibia E. Farag A.. (2024). Antimicrobial resistance of *Pseudomonas aeruginosa*: navigating clinical impacts, current resistance trends, and innovations in breaking therapies. Front. Microbiol. 15:1374466. doi: 10.3389/fmicb.2024.1374466 38646632 PMC11026690

[ref9002] Feliziani S. Marvig R. L. Luján A. M. Moyano A. J. Di Rienzo J. A. Krogh Johansen H.. (2014). Coexistence and Within-Host Evolution of Diversified Lineages of Hypermutable Pseudomonas aeruginosa in Long-term Cystic Fibrosis Infections. PLoS Genet. 10:e1004651. doi: 10.1371/journal.pgen.1004651 25330091 PMC4199492

[ref22] Folkesson A. Jelsbak L. Yang L. Johansen H. K. Ciofu O. Hoiby N.. (2012). Adaptation of *Pseudomonas aeruginosa* to the cystic fibrosis airway: an evolutionary perspective. Nat. Rev. Microbiol. 10, 841–851. doi: 10.1038/nrmicro2907 23147702

[ref23] Fritz S. A. Purvis A. (2010). Selectivity in mammalian extinction risk and threat types: a new measure of phylogenetic signal strength in binary traits. Conserv. Biol. 24, 1042–1051. doi: 10.1111/j.1523-1739.2010.01455.x 20184650

[ref24] Gibson R. L. Burns J. L. Ramsey B. W. (2003). Pathophysiology and Management of Pulmonary Infections in cystic fibrosis. Am. J. Respir. Crit. Care Med. 168, 918–951. doi: 10.1164/rccm.200304-505SO 14555458

[ref25] Hefti M. H. Françoijs K. J. de Vries S. C. Dixon R. Vervoort J. (2004). The PAS fold. A redefinition of the PAS domain based upon structural prediction. Eur. J. Biochem. 271, 1198–1208. doi: 10.1111/j.1432-1033.2004.04023.x 15009198

[ref26] Hoboth C. Hoffmann R. Eichner A. Henke C. Schmoldt S. Imhof A.. (2009). Dynamics of adaptive microevolution of hypermutable *Pseudomonas aeruginosa* during chronic pulmonary infection in patients with cystic fibrosis. J. Infect. Dis. 200, 118–130. doi: 10.1086/599360 19459782

[ref9003] Hogardt M. Hoboth C. Schmoldt S. Henke C. Bader L. Heesemann J. (2007). Stage-specific adaptation of hypermutable Pseudomonas aeruginosa isolates during chronic pulmonary infection in patients with cystic fibrosis. J. Infect. Dis. 195, 70–80. doi: 10.1086/509821 17152010

[ref27] Hsiao Y. M. Liu Y. F. Fang M. C. Song W. L. (2011). XCC2731, a GGDEF domain protein in *Xanthomonas campestris*, is involved in bacterial attachment and is positively regulated by Clp. Microbiol. Res. 166, 548–565. doi: 10.1016/j.micres.2010.11.003 21237626

[ref28] Hyatt D. Chen G. L. LoCascio P. F. Land M. L. Larimer F. W. Hauser L. J. (2010). Prodigal: prokaryotic gene recognition and translation initiation site identification. BMC Bioinformatics 11:119. doi: 10.1186/1471-2105-11-119 20211023 PMC2848648

[ref29] Iwańska A. Trafny E. A. Czopowicz M. Augustynowicz-Kopeć E. (2023). Phenotypic and genotypic characteristics of *Pseudomonas aeruginosa* isolated from cystic fibrosis patients with chronic infections. Sci. Rep. 13:11741. doi: 10.1038/s41598-023-39005-9 37474574 PMC10359326

[ref30] Jeukens J. Boyle B. Kukavica-Ibrulj I. Ouellet M. M. Aaron S. D. Charette S. J.. (2014). Comparative genomics of isolates of a *Pseudomonas aeruginosa* epidemic strain associated with chronic lung infections of cystic fibrosis patients. PLoS One. 9:e87611. doi: 10.1371/journal.pone.0087611 24505294 PMC3914812

[ref31] Jochumsen N. Marvig R. Damkiær S. Marvig R. L. Jensen R. L. Paulander W.. (2016). The evolution of antimicrobial peptide resistance in *Pseudomonas aeruginosa* is shaped by strong epistatic interactions. Nat. Commun. 7:13002. doi: 10.1038/ncomms13002 27694971 PMC5494192

[ref32] Kenna D. T. Doherty C. J. Foweraker J. Macaskill L. Barcus V. A. Govan J. R. W. (2007). Hypermutability in environmental *Pseudomonas aeruginosa* and in populations causing pulmonary infection in individuals with cystic fibrosis. Microbiology 153, 1852–1859. doi: 10.1099/mic.0.2006/005082-0 17526842

[ref33] Klockgether J. Miethke N. Kubesch P. Bohn Y. S. Brockhausen I. Cramer N.. (2013). Intraclonal diversity of the *Pseudomonas aeruginosa* cystic fibrosis airway isolates TBCF10839 and TBCF121838: distinct signatures of transcriptome, proteome, metabolome, adherence and pathogenicity despite an almost identical genome sequence. Environ. Microbiol. 15, 191–210. doi: 10.1111/j.1462-2920.2012.02842.x 22882573

[ref34] Koehorst J. J. Van Dam J. C. J. Saccenti E. Martins Dos Santos V. A. P. Suarez-Diez M. Schaap P. J. (2018). SAPP: functional genome annotation and analysis through a semantic framework using FAIR principles. Bioinformatics 34, 1401–1403. doi: 10.1093/bioinformatics/btx767 29186322 PMC5905645

[ref35] Köser C. U. Ellington M. J. Peacock S. J. (2014). Whole-genome sequencing to control antimicrobial resistance. Trends Genet. 30, 401–407. doi: 10.1016/j.tig.2014.07.003 25096945 PMC4156311

[ref36] Kothari A. Kherdekar R. Mago V. Uniyal M. Mamgain G. Kalia R. B.. (2023). Age of antibiotic resistance in MDR/XDR clinical pathogen of *Pseudomonas aeruginosa*. Pharmaceuticals 16:1230. doi: 10.3390/ph16091230 37765038 PMC10534605

[ref37] Lamut A. Peterlin Mašič L. Kikelj D. Tomašič T. (2019). Efflux pump inhibitors of clinically relevant multidrug resistant bacteria. Med. Res. Rev. 39, 2460–2504. doi: 10.1002/med.21591 31004360

[ref38] Lechtzin N. John M. Irizarry R. Merlo C. Diette G. B. Boyle M. P. (2006). Outcomes of adults with cystic fibrosis infected with antibiotic-resistant *Pseudomonas aeruginosa*. Respiration 73, 27–33. doi: 10.1159/000087686 16113513

[ref39] Lobb B. Tremblay B. J. M. Moreno-Hagelsieb G. Doxey A. C. (2020). An assessment of genome annotation coverage across the bacterial tree of life. Microb. Genom. 6:e000341. doi: 10.1099/mgen.0.000341 32124724 PMC7200070

[ref40] Mahmood Y. Jamshidi S. Mark Sutton J. (2016). Current advances in developing inhibitors of bacterial multidrug efflux pumps. Curr. Med. Chem. 23, 1062–1081. doi: 10.2174/0929867323666160304150522 26947776 PMC5425656

[ref41] Manrubia S. Cuesta J. A. Aguirre J. Ahnert S. E. Altenberg L. Cano A. V.. (2021). From genotypes to organisms: state-of-the-art and perspectives of a cornerstone in evolutionary dynamics. Phys Life Rev 38, 55–106. doi: 10.1016/j.plrev.2021.03.004 34088608

[ref42] Martínez-Gallardo M. J. Villicaña C. Yocupicio-Monroy M. Alcaraz-Estrada S. L. Salazar-Salinas J. Mendoza-Vázquez O. F.. (2024). Comparative genomic analysis of *Pseudomonas aeruginosa* strains susceptible and resistant to carbapenems and aztreonam isolated from patients with healthcare-associated infections in a Mexican hospital. Mol. Gen. Genomics. 299:29. doi: 10.1007/s00438-024-02122-9 38472486

[ref43] Marvig R. L. Johansen H. K. Molin S. Jelsbak L. (2013). Genome analysis of a transmissible lineage of *Pseudomonas aeruginosa* reveals Pathoadaptive mutations and distinct evolutionary paths of Hypermutators. PLoS Genet. 9:e1003741. doi: 10.1371/journal.pgen.1003741 24039595 PMC3764201

[ref44] Mayer-Hamblett N. Rosenfeld M. Gibson R. L. Ramsey B. W. Kulasekara H. D. Retsch-Bogart G. Z.. (2014). *Pseudomonas aeruginosa* in vitro phenotypes distinguish cystic fibrosis infection stages and outcomes. Am. J. Respir. Crit. Care Med. 190, 289–297. doi: 10.1164/rccm.201404-0681OC 24937177 PMC4226041

[ref45] Mena A. Smith E. E. Burns J. L. Speert D. P. Moskowitz S. M. Perez J. L.. (2008). Genetic adaptation of *Pseudomonas aeruginosa* to the airways of cystic fibrosis patients is catalyzed by hypermutation. J. Bacteriol. 190, 7910–7917. doi: 10.1128/JB.01147-08 18849421 PMC2593214

[ref46] Metzger G. A. Ridenhour B. J. France M. Gliniewicz K. Millstein J. Settles M. L.. (2022). Biofilms preserve the transmissibility of a multi-drug resistance plasmid. NPJ Biofilms Microbiomes 8:95. doi: 10.1038/s41522-022-00357-1 36481746 PMC9732292

[ref47] Mikkelsen H. Hui K. Barraud N. Filloux A. (2013). The pathogenicity island encoded PvrSR/RcsCB regulatory network controls biofilm formation and dispersal in *Pseudomonas aeruginosa* PA14. Mol. Microbiol. 89, 450–463. doi: 10.1111/mmi.12287 23750818 PMC3842833

[ref48] Mistry J. Chuguransky S. Williams L. Qureshi M. Salazar G. A. Sonnhammer E. L. L.. (2021). Pfam: the protein families database in 2021. Nucleic Acids Res. 49, D412–D419. doi: 10.1093/nar/gkaa913 33125078 PMC7779014

[ref49] Murray C. J. Ikuta K. S. Sharara F. Swetschinski L. Robles Aguilar G. Gray A.. (2022). Global burden of bacterial antimicrobial resistance in 2019: a systematic analysis. Lancet 399, 629–655. doi: 10.1016/S0140-6736(21)02724-0 35065702 PMC8841637

[ref50] Nordmann P. Poirel L. (2019). Epidemiology and diagnostics of Carbapenem resistance in gram-negative Bacteria. Clin. Infect. Dis. 69, S521–S528. doi: 10.1093/cid/ciz824 31724045 PMC6853758

[ref51] O’Neill J. (2016). Tackling drug-resistant infections globally: final report and recommendation the review on antimicrobial resistance chaired by Jim O’Neill. Available at: https://amr-review.org/ (Accessed July 31, 2025).

[ref52] O’Toole G. A. (2011). Microtiter dish biofilm formation assay. J. Vis. Exp. doi: 10.3791/2437 PMC3182663 21307833

[ref53] Oliver A. Cantòn R. Pilar C. Baquero F. Blàsquez J. (2000). High frequency of hypermutable *Pseudomonas aeruginosa* in cystic fibrosis lung infection. Science 288, 1251–1253. doi: 10.1126/science.288.5469.1251 10818002

[ref54] Oliver A. Rojo-Molinero E. Arca-Suarez J. Beşli Y. Bogaerts P. Cantón R.. (2024). *Pseudomonas aeruginosa* antimicrobial susceptibility profiles, resistance mechanisms and international clonal lineages: update from ESGARS-ESCMID/ISARPAE group. Clin. Microbiol. Infect. 30, 469–480. doi: 10.1016/j.cmi.2023.12.026 38160753

[ref55] Pommerenke C. Müsken M. Becker T. Dötsch A. Klawonn F. Haussler S. (2010). Global genotype-phenotype correlations in *pseudomonas aeruginosa*. PLoS Pathog. 6:e1001074. doi: 10.1371/journal.ppat.1001074 20865161 PMC2928780

[ref56] Reichhardt C. (2023). The *Pseudomonas aeruginosa* biofilm matrix protein CdrA has similarities to other fibrillar adhesin proteins. J. Bacteriol. 205:e00019-23. doi: 10.1128/jb.00019-23 37098957 PMC10210978

[ref57] Renwick M. J. Simpkin V. Mossialos E. (2016). Targeting innovation in antibiotic drug discovery and development: The need for a One Health--One Europe--One World Framework. Europe: World Health Organization. Regional Office for Europe. 28806044

[ref58] Richardson E. J. Watson M. (2013). The automatic annotation of bacterial genomes. Brief. Bioinform. 14, 1–12. doi: 10.1093/bib/bbs007 22408191 PMC3548604

[ref59] Roberts D. W. (2010). “Ordination and Multivariate Analysis for Ecology: package ‘labdsv,’” in R: A Language and Environment for Statistical Computing. eds. Roberts D. W. David M. Roberts W..

[ref60] Roca I. Akova M. Baquero F. Carlet J. Cavaleri M. Coenen S.. (2015). The global threat of antimicrobial resistance: science for intervention. New Microbes New Infect 6, 22–29. doi: 10.1016/j.nmni.2015.02.007 26029375 PMC4446399

[ref61] Rojas L. J. Yasmin M. Benjamino J. Marshall S. M. DeRonde K. J. Krishnan N. P.. (2022). Genomic heterogeneity underlies multidrug resistance in *Pseudomonas aeruginosa*: a population-level analysis beyond susceptibility testing. PLoS One 17:e0265129. doi: 10.1371/journal.pone.0265129 35358221 PMC8970513

[ref62] Rossi E. La Rosa R. Bartell J. A. Marvig R. L. Haagensen J. A. J. Sommer L. M.. (2021). *Pseudomonas aeruginosa* adaptation and evolution in patients with cystic fibrosis. Nat. Rev. Microbiol. 19, 331–342. doi: 10.1038/s41579-020-00477-5 33214718

[ref63] Sahayarayan J. J. Thiyagarajan R. Prathiviraj R. K T. Rajan K. S. Manivannan P.. (2024). Comparative genome analysis reveals putative and novel antimicrobial resistance genes common to the nosocomial infection pathogens. Microb. Pathog. 197:107028. doi: 10.1016/j.micpath.2024.107028 39426637

[ref64] Seemann T. (2015). Snippy-rapid haploid variant calling and core SNP phylogeny: GitHub.

[ref65] Sousa A. Pereira M. (2014). *Pseudomonas aeruginosa* diversification during infection development in cystic fibrosis lungs—a review. Pathogens. 3, 680–703. doi: 10.3390/pathogens3030680 25438018 PMC4243435

[ref66] Thöming J. G. Häussler S. (2022). *Pseudomonas aeruginosa* is more tolerant under biofilm than under planktonic growth conditions: a multi-isolate survey. Front. Cell. Infect. Microbiol. 28:851784. doi: 10.3389/fcimb.2022.851784 PMC8920030 35295755

[ref67] Vetrivel A. Ramasamy M. Vetrivel P. Natchimuthu S. Arunachalam S. Kim G.-S.. (2021). *Pseudomonas aeruginosa* biofilm formation and its control. Biologics 1, 312–336. doi: 10.3390/biologics1030019

[ref68] Vincent A. T. (2024). Bacterial hypothetical proteins may be of functional interest. Front. Bacteriol. 3:1334712. doi: 10.3389/fbrio.2024.1334712

[ref69] Waddington C. Carey M. E. Boinett C. J. Higginson E. Veeraraghavan B. Baker S. (2022). Exploiting genomics to mitigate the public health impact of antimicrobial resistance. Genome Med. 14. doi: 10.1186/s13073-022-01020-2 PMC8849018 35172877

[ref70] Wei G. Palalay J. S. Sanfilippo J. E. Yang J. Q. (2025). Flagellum-driven motility enhances *Pseudomonas aeruginosa* biofilm formation by altering cell orientation. Appl. Environ. Microbiol. 1, e00821–e00825. doi: 10.1128/aem.00821-25 PMC12285247 40607807

[ref71] Weimann A. Dinan A. M. Ruis C. Bernut A. Pont S. Brown K.. (2024). Evolution and host-specific adaptation of *Pseudomonas aeruginosa*. Science 385:eadi0908. doi: 10.1126/science.adi0908 38963857 PMC7618370

[ref72] WHO (2020) GLASS whole-genome sequencing for surveillance of antimicrobial resistance

[ref73] Winstanley C. O’Brien S. Brockhurst M. A. (2016). *Pseudomonas aeruginosa* evolutionary adaptation and diversification in cystic fibrosis chronic lung infections. Trends Microbiol. 24, 327–337. doi: 10.1016/j.tim.2016.01.008 26946977 PMC4854172

[ref74] Yu Z. Zhang W. Yang H. Chou S. H. Galperin M. Y. He J. (2023). Gas and light: triggers of c-di-GMP-mediated regulation. FEMS Microbiol. Rev. 47:fuad034. doi: 10.1093/femsre/fuad034 37339911 PMC10505747

